# Upregulation of AMPK by 4‐O‐methylascochlorin promotes autophagy via the HIF‐1α expression

**DOI:** 10.1111/jcmm.13933

**Published:** 2018-10-19

**Authors:** Ji‐Young Seok, Yun‐Jeong Jeong, Soon‐Kyung Hwang, Cheorl‐Ho Kim, Junji Magae, Young‐Chae Chang

**Affiliations:** ^1^ Research Institute of Biomedical Engineering and Department of Medicine Catholic University of Daegu School of Medicine Daegu Korea; ^2^ Department of Biological Science Sungkyunkwan University Suwon Kyunggi Korea; ^3^ Magae Bioscience Institute Tsukuba Japan

**Keywords:** 4‐O‐methylascochlorin, AMPK, autophagy, BNIP3, HIF‐1α

## Abstract

4‐O‐methylascochlorin (MAC) is a derivative of ascochlorin, a prenyl‐phenol compound antibiotic isolated from the fungus *Ascochyta viciae*. MAC induces caspase/poly (ADP‐ribose) polymerase‐mediated apoptosis in leukemia cells. However, the effects of MAC on autophagy in cancer cells and the underlying molecular mechanisms remain unknown. Here, we show that MAC induces autophagy in lung cancer cells. MAC significantly induced the expression of autophagy marker proteins including LC3‐II, Beclin1, and ATG7. MAC promoted AMP‐activated protein kinase (AMPK) phosphorylation and inhibited the phosphorylation of mammalian target of rapamycin (mTOR) and its downstream signalling proteins P70S6K and 4EBP1. The AMPK activator AICAR upregulated LC3‐II expression through the AMPK/mTOR pathway similar to the effects of MAC. MAC‐induced LC3‐II protein expression was slightly reduced in AMPK siRNA transfected cells. MAC upregulated hypoxia‐inducible factor‐1α (HIF‐1α) and BNIP3, which are HIF‐1α‐dependent autophagic proteins. Treatment with CoCl_2_, which mimics hypoxia, induced autophagy similar to the effect of MAC. The HIF‐1α inhibitor YC‐1 and HIF‐1α siRNA inhibited the MAC‐induced upregulation of LC3‐II and BNIP3. These results suggest that MAC induces autophagy via the AMPK/mTOR signalling pathway and by upregulating HIF‐1α and BNIP3 protein expression in lung cancer cells.

## 1 INTRODUCTION

Human lung cancer is a malignancy of the bronchus and alveoli. The mortality of lung cancer is continuously increasing worldwide.^1^ There are various treatment modalities for lung cancer including surgery, radiotherapy, chemotherapy, and molecular targeted therapy.^2^ However, the survival rate of lung cancer patients is poor because lung cancer is difficult to detect in its early stage, and is a very aggressive and invasive cancer. The rapid growth of lung cancer remains a serious obstacle to the success of therapy.

Apoptosis and autophagy are programmed cell death and progressive cell death during which a cell uses a specificity intracellular system to kill itself. Autophagy is a self‐degradation process of intracellular organelles by which cytoplasmic components are recycled during starvation, under stressful conditions, and in response to cell growth alterations.^3^ Autophagy involves the formation of autophagosomes through the extension of the phagophore, which generates vesicles that enclose damaged organelles within a double membrane before fusing with lysosomes.^4^ It is a highly regulated process that depends on the interaction of approximately 30 autophagy‐related genes (ATG) present from yeasts to mammals. In the ATG family, ATG8, also known as LC3, plays an essential role in the formation of the autophagosome.^5^ It is localized in the autophagosomal membrane and is the main protein involved in the autophagy process.^6^ The activation of LC3 is controlled by ATG4, which converts LC3 to LC3‐I by removing its C‐terminus.^5^ LC3‐I, which has an exposed glycine residue, is transformed into LC3‐II by ATG3 through conjugation with phosphatidylethanolamine (PE), and LC3‐II is recruited to the autophagosomal membrane through the action of the ATG12/ATG5/ATG16 complex.^7^, ^8^, ^9^

Autophagy is induced in response to stresses such as the hypoxic (low‐oxygen) condition. During hypoxia stress, autophagy can be induced by hypoxia‐inducible factor‐1α (HIF‐1α), an important transcription factor that functions in cell survival in response to hypoxia and according to the extent of exposure.^10^ In addition to hypoxia stress, decreased energy and glucose supply induce autophagy via HIF‐1α independent signalling pathways. These signalling mechanisms involve the AMP‐activated protein kinase (AMPK) signal, which responds to nutritional stress.^11^ A cascade of AMPK‐triggered phosphorylation events eventually inhibits the ATP‐consuming anabolic processes, stimulates the ATP‐producing catabolic processes, and initiates autophagy.^12^

Ascochlorin (ASC) is a prenyl‐phenol compound isolated from the fungus *Ascochyta viciae*. ASC‐related compounds were originally characterized as antiviral antibiotics.^13^ ASC functions as an antibiotic,^14^ promotion hypolipidemic activity,^15^ anti‐diabetic,^16^ immunomodulation.^17^ In addition to ASC, ASC‐related derivatives like 4‐O‐carboxymethylascochlorin (AS‐6) and 4‐O‐methylascochlorin (MAC)^16^ have been reported to be therapeutic reagents for various physiological events.^18^ Among the derivatives of ASC, MAC‐induced apoptotic effects through caspase/poly (ADP‐ribose) polymerase‐mediated apoptosis in leukemia cells.^13^ However, the effects of MAC on autophagy in cancer cells and the underlying molecular mechanisms have not been investigated to date.

In this study, the effects of MAC on autophagy and the mechanism underlying MAC‐mediated autophagy in lung cancer cells were investigated. Our results showed that MAC‐induced autophagosome formation by upregulating LC3‐II, Beclin1, and ATG7, and the effects of MAC on autophagy were related to the regulation of HIF‐1α and the AMPK/mammalian target of rapamycin (mTOR) signalling pathway.

## 2 MATERIALS AND METHODS

### 2.1 Cells culture and materials

Human non‐small lung cancer cell lines A549, H1793, and H23 cells were obtained from the American Type Culture Collection (Rockville, MD, USA). Cells were cultured in RPMI 1640 (Thermo Scientific, Logan, UT, USA), containing 10% of fetal bovine serum and 1% of antibiotic mixture. These were incubated at 5% of CO_2_, 37°C. MAC was supplied Chugai Pharmaceutical (Tokyo, Japan).

### 2.2 Cell viability assay

Human non‐small lung cancer cell lines were seeded in 96‐well plates at 2 × 10^4^ cells/well in RPMI 1640 media, and allowed to attach for 24 hours. New media containing different concentration treat of MAC and incubate for 24 hours. 3‐[4,5‐Dimethylthiazol‐2‐yl]‐2,5‐diphenyltetra‐zolium bromide (MTT) (Roche Molecular Biochemicals, Indianapolis, IN, USA) was added to each well. The amount of formazan deposits was quantified according to the supplier's protocol after 4 hours of incubation with MTT assay in 5% of CO_2_ at 37°C.

### 2.3 Morphology

Cells were cultured in 60 mm dish and incubated until they reached 60% confluence. Then, media were changed fresh RPMI 1640 media and treat MAC for 12 hours, and cells were photographed.

### 2.4 Western blot analysis

Cells lysates were prepared by lysis buffer [50 mmol/L Tris, 150 mmol/L NaCl, 5 mmol/L EDTA, 1 M DTT, 1% NP‐40, 100 μmol/L phenylmethylsulfonyl fluoride, 20 μmol/L aprotinin, and 20 μmol/L leupeptin, adjusted to (pH 8.0)]. Total proteins were electrotransferred to Immobilon‐P membranes (Millipore, Billerica, MA, USA). Detection of specific proteins was carried out with an enhanced chemiluminescence Western blotting kit, following the manufacturer's instructions (Amersham, Piscataway, NJ, USA). To determine the activations of HIF‐1α nuclear extracts of cells were subjected to describe as follows. Cells were suspended in tubes with 0.4 mL of lysis buffer [10 mmol/L HEPES (pH 7.9), 10 mmol/L KCl, 0.1 mmol/L EDTA, 0.1 mmol/L EGTA, 1 mmol/L DTT, 0.5 mmol/L PMSF, 2 μg/mL leupeptin, and 2 μg/mL aprotinin]. Cells were then allowed to swell on ice for 5 minutes and 25 μL of 10% Nonidet P‐40 was added. Homogenates were centrifuged at 4°C for 1 minute at 12 204 *g*. The nuclear pellets were resuspended in 50 μL of ice‐cold nuclear extraction buffer containing [20 mmol/L HEPES (pH 7.9), 0.4 M NaCl, 1 mmol/L EDTA, 1 mmol/L EGTA, 1 mmol/L DTT, 1 mmol/L PMSF, 2 μg/mL leupeptin, and 2 μg/mL aprotinin], and incubated on ice for 15 minutes with intermittent mixing. Nuclear extracts were then centrifuged at 4°C for 5 minutes at 10 000 rpm and supernatants were either used immediately. Specific antibodies were purchased from Cell Signaling Technology (Danvers, MA, USA) for LC3, p62, ATG7, transcription factor EB (TFEB), p70S6K, AMPK, phosphorylation of AMPK and Santa Cruz Biotechnology (Dallas, TX, USA) for BNIP3, Beclin1, HIF‐1α, phosphorylation of p70S6K, and phosphorylation of 4EBP1.

### 2.5 Acridine orange staining

Acridine orange (1 μg/mL) was added to previously treat‐cells for 1 hour at 37°C and fixed with 4% of Formaldehyde for 1 minute. Then, cells were washed three times with PBS containing 0.05% v/v Tween‐20 and 1% w/v BSA. Coverslips were mounted by using fluorescence mounting medium. Images of cells were observed using confocal microscope.

### 2.6 siRNA transfection

For transfection experiments with siRNA directed against AMPK, HIF‐1α, and ATG7 the medium for A549 and H23 cell was changed to fresh RPMI, and the cell was transfected for 24 hours with 10 μmol/L of siRNA by Trans IT‐TKO (Mirusbio, Madison, WI, USA) according to the manufacturer's instruction. After 24 hours, the medium was changed to fresh serum‐free RPMI, cell was treated with TPA or 24 hours and total protein in the cell was extracted for Western blot.

### 2.7 Immunofluorescence microscopy

Cells were cultured and treated on poly‐l‐lysine‐coated coverslips before being fixed in 100% ethanol for 1 minute at room temperature. After two washes with PBS, cells were permeabilized with 0.2% w/v Triton X‐100 in PBS for 5 minutes. After two washes with PBS, cells were blocked with 10% v/v normal goat serum in PBS for 1 hour in a humidified chamber. Cells were then incubated with primary antibodies LC3 (1:100, diluted in PBS containing 2% v/v normal goat serum) for 1 hour. The cells were then washed three times before being incubated with FITC, tetramethylrhodamine isothiocyanate‐conjugated secondary antibody rabbit immunoglobulin (1:100, diluted in PBS containing 2% v/v normal goat serum) for 1 hour, and five times were washed with PBS containing 0.05% v/v Tween‐20 and 1% w/v BSA. Then, cells were incubated with DAPI for 3 minutes at 37°C incubation. Finally, the cells were washed three times with PBS. Coverslips were mounted with 90% of glycerol and sealed with Mounting Media. Slides were examined and scanned on a fluorescence microscope.

### 2.8 GFP‐LC3 assay

After transient transfection with GFP‐LC3 plasmid (EGFP‐LC3 plasmid was a gift from Karla Kirkegaard; Addgene plasmid #11546), A549 cells were cultured on poly‐l‐lysine‐coated coverslips. After 24 hours, cells were then transfected with 3 μg plasmid of GFP‐LC3 using transfection reagent Lipofectamine 2000 (Invitrogen; Thermo Fisher Scientific, Inc.) according to the manufacturer's instructions. After 24 hours medium incubation, cells were treated with MAC, CoCl_2_, and EBSS for 24 hours and fixed with 4% of formaldehyde for 5 minutes and three times washed with PBS. Then, cells were incubated with DAPI for 3 minutes at 37°C. Finally, the cells were washed five times with PBS. Coverslips were mounted by using fluorescence mounting medium. Slides were examined and observed of GFP‐LC3 puncta on a fluorescence microscope.

### 2.9 Statistical analysis

All in vitro results are representative of at least three independent experiments performed in triplicate. *P < 0.05, statistically significant between experimental and control values. Significance of differences between experimental and control values was calculated using ANOVA with Newman–Keuls multicomparison test.

## 3 RESULTS

### 3.1 Effects of ASC and ASC derivatives on autophagosome formation in A549 lung cancer cells

**Figure 1:**
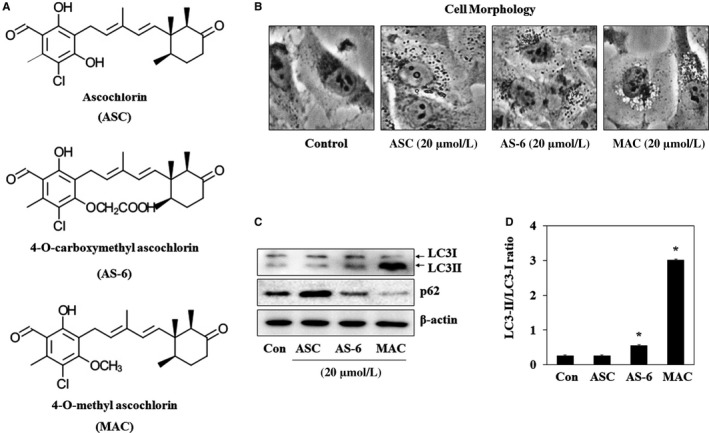
Effects of ASC and ASC‐derivatives on autophagy in A549 lung cancer cells. (A) Chemical structure of ASC, AS‐6, and MAC. (B) A549 cells were treated with 20 μmol/L ASC, AS‐6, and MAC for 12 h, and cell morphology was observed under a microscope. (C) Cells were incubated with 20 μmol/L ASC, AS‐6, and MAC for 12 h and subjected to western blot analysis with antibodies against LC3 and p62. β‐actin was used as a loading control. (D) Densitometric analysis of LC3‐II levels relative to those of LC3‐I was performed using ImageJ. The data represent the mean ± SE of three independent experiments (*P < 0.05 vs control)

The typical morphological feature of autophagy is the formation of autophagosomes, which fuse with lysosomes and expose cargo to lysosomal enzymes, causing degradation of the contents.^19^ The effect of ASC and its derivatives AS‐6 and MAC on autophagosome formation (Figure 1A) was investigated by examining morphological changes of A549 lung cancer cells. Incubation of A549 cells with 20 μmol/L ASC, AS‐6, or MAC for 12 hours induced vesicle formation (Figure 1B). LC3 and p62 (also known as SQSTM1) are the key factors for the autophagy.^20^ Accumulation of LC3‐II allows assessment of autophagy initiation, whereas p62 is degraded by autophagy. The effect of the three chemicals on autophagosome formation was investigated by western blot analysis of LC3‐I/II and p62 expression. As shown in Figure 1C, AS‐6 and MAC but not ASC upregulated LC3‐II. AS‐6 and MAC increased the LC3‐II/LC3‐I ratio by approximately 3‐ and 10‐fold, respectively, whereas ASC had no effect on the LC3‐II/LC3‐I ratio. MAC also completely degraded p62. These data suggest that MAC induces autophagy more effectively than the other ASC derivatives. Therefore, the mechanism of MAC‐induced autophagy was analysed in this study.

### 3.2 MAC triggered autophagosome formation in various lung cancer cell lines

**Figure 2:**
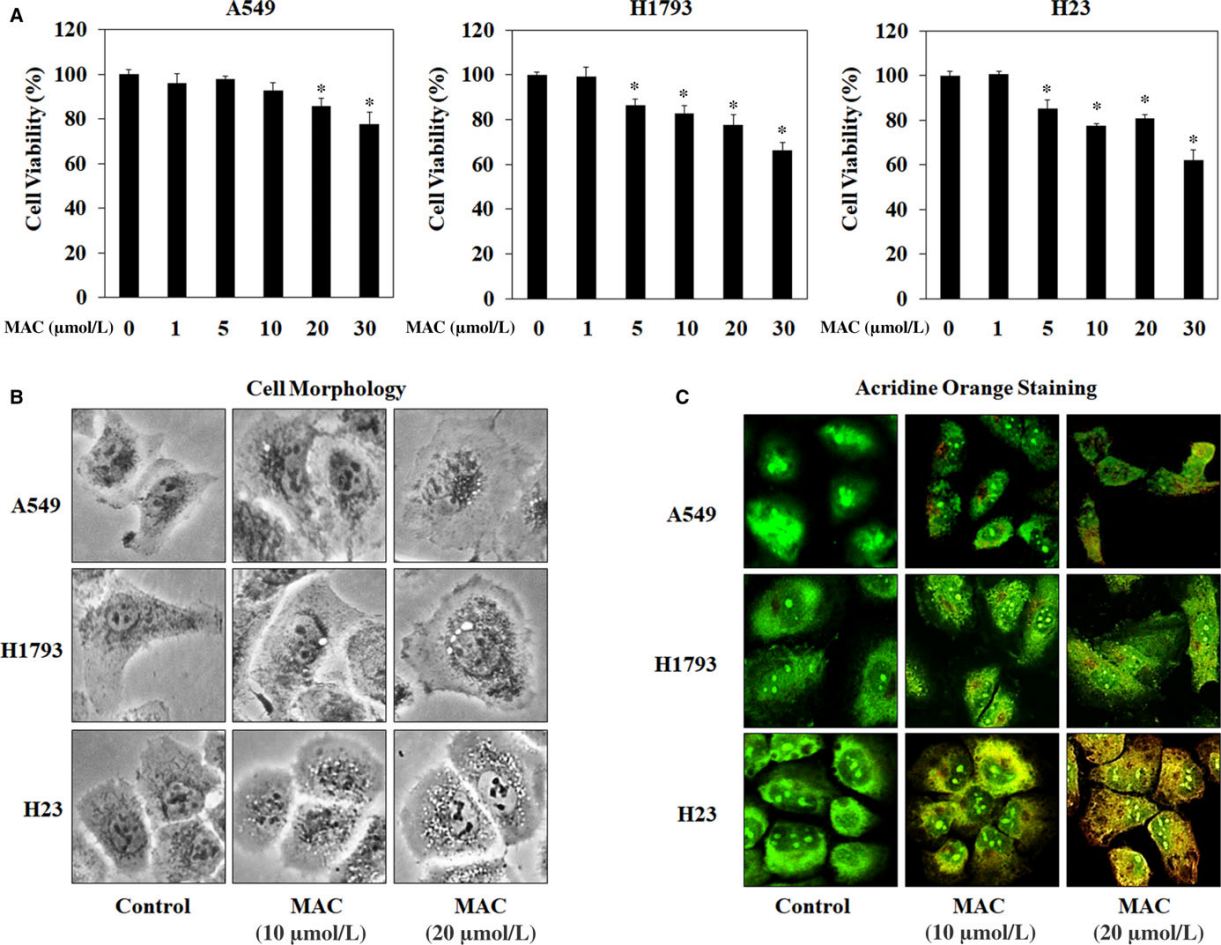
MAC induces autophagosome vesicle formation in lung cancer cells. (A) Cells were treated with the indicated concentrations of MAC for 24 h, after which cell viability was measured using the MTT assay. (B) Cells were treated with MAC at 10 and 20 μmol/L for 24 h, and cell morphology was observed under a microscope. (C) Cells were treated with MAC for 24 h and then stained with acridine orange for autophagosome detection. Cells were visualized using a confocal laser microscope. The data represents mean ± SE of three independent experiments (*P < 0.05 vs control)

The effects of MAC on autophagy were examined in three lung cancer cell lines: A549, H1793, and H23. Before investigating the pharmacological effect of MAC on autophagy, the cytotoxic effect of MAC was examined using the MTT assay. The results showed no obvious reduction in A549 cell viability after treatment with MAC at doses lower than 10 μmol/L. MAC at 20 μmol/L decreased cell viability by approximately 20%, and a cytotoxic effect was observed at 30 μmol/L in H1793 and H23 cells (Figure 2A). Based on these results, a concentration of <20 μmol/L MAC was used in subsequent experiments. The effect of MAC treatment on the morphology of the three lung cancer cell lines was examined. As shown in Figure 2B, MAC increased vesicle formation in a dose‐dependent manner (at 10 and 20 μmol/L). To detect the development of autophagosome vesicles, cells were stained with acridine orange. A549, H1793, and H23 cells were cultured in the presence of different concentrations of MAC for 24 hours and then stained. The results showed that MAC triggered autophagosome vesicle formation in lung cancer cells (Figure 2C), suggesting that MAC induces autophagy by promoting the formation of autophagosomes.

### 3.3 MAC‐induced autophagy‐related gene expression

**Figure 3:**
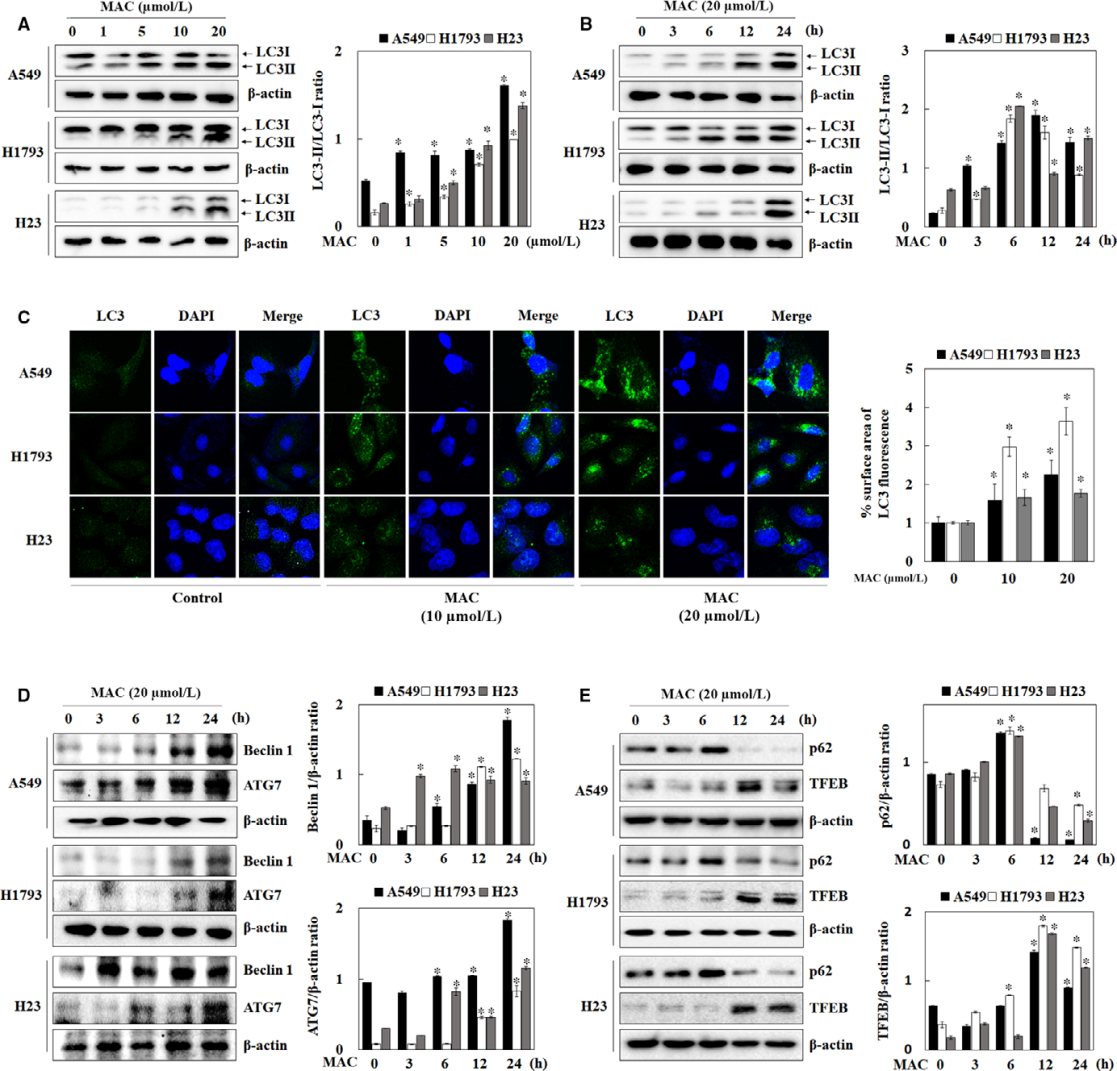
MAC induces autophagy by increasing LC3 expression. (A and B) Cells were treated with the indicated concentrations of MAC for 12 h and 20 μmol/L of MAC for various periods of time. Western blot analysis was performed with antibodies against LC3. β‐actin was used as a loading control. (C) Cells were seeded onto glass coverslips and cultured overnight. Media were replaced with the indicated concentrations of MAC for 12 h, and then the cells were stained for LC3 (green fluorescence) and DAPI (blue). Cells were visualized using a confocal laser microscope. Densitometric analysis of LC3 levels (green fluorescence) was performed using ImageJ. (D and E) Cells were treated with 20 μmol/L MAC for various periods of time and analysed by western blotting with antibodies against Beclin1, ATG7, p62, and TFEB. β‐actin was used as a loading control. Densitometric analysis of each protein ratio was performed using ImageJ. The data represent mean ± SE of three independent experiments (*P < 0.05 vs control and ^#^ *P* < 0.05 vs MAC)

To determine whether autophagy was induced by MAC in lung cancer cells, the expression of LC3‐I/II, a key autophagy marker protein, was measured by western blotting. As shown in Figure 3A and B, MAC upregulated LC3‐II in a dose‐ and time‐dependent manner in A549 and H1793 cells. In H23 cells, MAC significantly increased the expression of both LC3‐I and II. To confirm the relationship between LC3‐II expression and autophagosome formation in response to MAC treatment, immunofluorescence was performed using an LC3 antibody. Cells treated with MAC had higher expression of LC3 than untreated cells (Figure 3C). Morphological analysis showed that MAC promoted the accumulation of LC3 around the nucleus. These results indicate that MAC‐induced autophagy by upregulating LC3 expression. The human Beclin1 gene is the mammalian homolog of the yeast ATG6/Vps30 gene, which plays an important role in autophagosome formation and autophagy activation.^21^ ATG proteins have been studied for their roles in autophagosome formation and maturation.^22^ We showed that MAC upregulated Beclin1 and ATG7 in a time‐dependent manner (Figure 3D). These results suggest that MAC promotes autophagy by upregulating LC3‐II, Beclin1, and ATG7 protein expression in lung cancer cells. We next evaluated changes in p62 and TFEB expression. p62 is selectively bind to LC3 and degraded by lysosome in the final stages of the autophagy process.^23^ The expression of p62 was increased at 6 hours after treatment with MAC. However, it began to decline at 12 hours compared with those in untreated (Figure 3E). TFEB, which is a regulator of autophagy to lysosomal biogenesis,^24^ also began to increase at 12 hours after MAC treatment. These results suggest that MAC promotes autolysosome by regulating p62 and TFEB protein expression in lung cancer cells.

### 3.4 MAC activated autophagy through the AMPK signalling pathway

**Figure 4:**
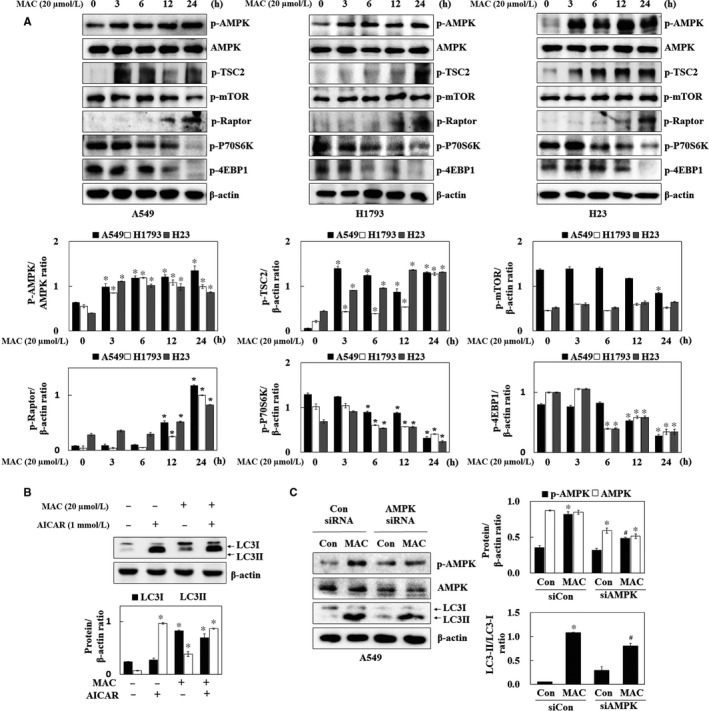
MAC induces the expression of autophagy‐associated proteins and AMPK/mTORC1 signalling pathway in lung cancer cells. (A) Cells were treated with 20 μmol/L MAC for various periods of time. The phosphorylated levels of AMPK, Raptor, TSC2, mTOR, P70S6K, and 4EBP1 were determined by western blot analysis. β‐actin was used as a loading control. (B) A549 cells were treated with MAC (20 μmol/L) and AICAR (1 mmol/L) analysed by western blotting for the LC3. β‐actin was used as a loading control. (C) A549 cells were transfected with control siRNA or AMPK siRNA, and then treated with MAC (20 μmol/L) for 12 h. Cells were analysed by western blotting for the phosphorylation of AMPK, AMPK, and LC3. β‐actin was used as a loading control. Densitometric analysis of each protein ratio was performed using ImageJ. The data represent mean ± SE of three independent experiments (*P < 0.05 vs control and ^#^ *P* < 0.05 vs MAC)

AMPK induces autophagy by stimulating the ULK1 and Beclin1 proteins mTOR, a downstream regulator of AMPK, stimulates protein synthesis and inhibits autophagy by disrupting the interaction between ULK1 and AMPK.^25^ We therefore examined the effect of MAC on the phosphorylation of AMPK and mTOR, the major direct signalling pathways of autophagy. MAC increased the phosphorylation of AMPK and tuberous sclerosis complex 2 (TSC2), an AMPK downstream regulator, in a time‐dependent manner in lung cancer cells (Figure 4A). However, MAC had no effect on the levels of phosphorylated mTOR compared with those in control H1793 and H23 cells. The phosphorylation of the regulatory associated protein mTOR (Raptor) at serine 792, which is phosphorylated by AMPK and leads to the inhibition of mTOR complex 1 (mTORC1), induced by MAC was therefore confirmed. MAC inhibited the phosphorylation of P70S6K and 4EBP1, the downstream factors of mTORC1, in a time‐dependent manner. These results indicate that mTORC1 downstream signalling was affected by MAC in a manner independent of the regulation of mTOR phosphorylation, and that MAC‐induced autophagy is involved in the AMPK/mTORC1 signalling pathway.

To confirm that MAC induced autophagy through the AMPK signalling pathway, LC3 expression was analysed in A549 cells treated with AICAR (AMPK activator). AICAR upregulated both LC3I and LC3II, whereas MAC‐induced LC3II expression compared with AICAR (Figure 4B). Transfection of cells with AMPK siRNA suppressed AMPK phosphorylation and expression and reduced MAC induced LC3‐II expression (Figure 4C). These results indicate that MAC‐induced autophagy is associated with the AMPK signalling pathway. However, the MAC‐induced LC3‐II expression was not completely suppressed in AMPK siRNA transfected cells, which suggest that the other signalling pathway might be involved in the MAC‐regulated autophagy.

### 3.5 MAC‐induced autophagy was related to hypoxia in lung cancer cells

**Figure 5:**
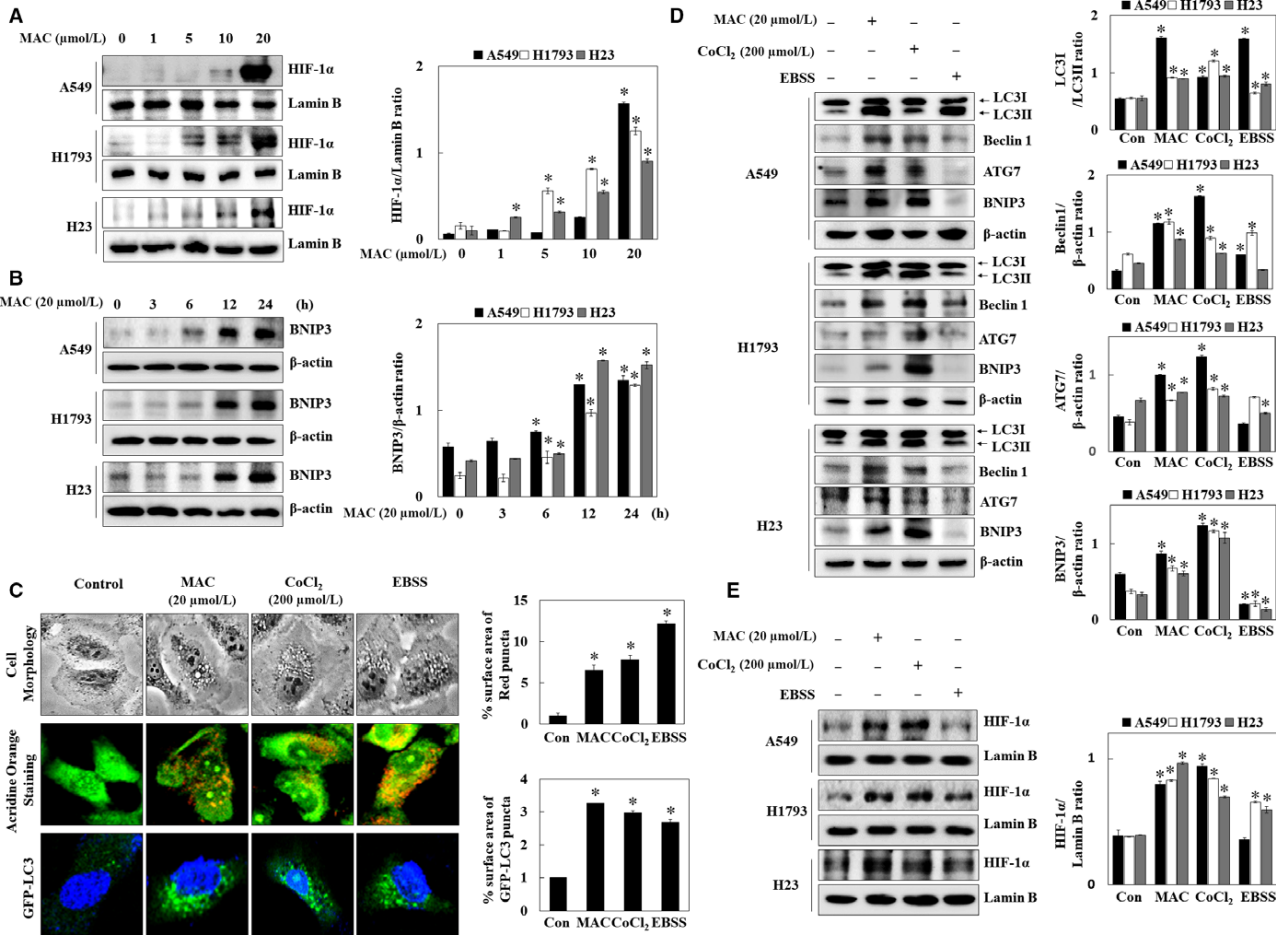
MAC induces the expression of BNIP3 and HIF‐1α translocation in lung cancer cells. (A) Cells were treated with the indicated concentrations of MAC for 6 h, and nuclear extracts were subjected to western blot analysis using antibodies against HIF‐1α. Lamin B was used as a nuclear loading control. (B) Cells were treated with 20 μmol/L MAC for various periods of time and analysed by western blotting for BNIP3. β‐actin was used as a loading control. (C) A549 cells were treated with MAC (20 μmol/L), CoCl_2_ (200 μmol/L), and EBSS media for 12 h. Cell morphology was observed under a microscope. Cells were stained with acridine orange for autophagosome formation and visualized by confocal microscopy. Cells stably transfected with GFP‐LC3 were treated with MAC (20 μmol/L), CoCl_2_ (200 μmol/L), and EBSS media for 24 h. GFP‐LC3 puncta was visualized by confocal microscopy. Densitometric analysis of each acridine orange (red), GFP‐LC3 (green), and DAPI (blue) were performed using ImageJ. (D) Cells were treated with MAC (20 μmol/L), CoCl_2_ (200 μmol/L), and EBSS media for 12 h and analysed by western blotting with antibodies against LC3, Beclin1, ATG7, and BNIP3. β‐actin was used as a loading control. (E) Cells were treated with MAC (20 μmol/L), CoCl_2_ (200 μmol/L), and EBSS media for 6 h, and nuclear extracts were subjected to western blot analysis using antibodies against HIF‐1α. Lamin B was used as a loading control. Densitometric analysis of each protein ratio was performed using ImageJ. The data represent mean ± SE of three independent experiments (*P < 0.05 vs control)

As HIF‐1 signalling responds to oxygen deficiency, AMPK, an energy deficiency sensor that responds to stressful environments, may play a fundamental role in cancer control and longevity.^26^ Therefore, we hypothesized that the MAC‐induced dysregulation of LC3‐II expression in AMPK siRNA transfected cells contributed to hypoxia‐mediated autophagy. To confirm this hypothesis, the effect of MAC on hypoxia‐dependent proteins was examined in lung cancer cells. Cells were treated with various concentrations of MAC for 6 hours, and the nuclear translocation of HIF‐1α was analysed (Figure 5A). MAC significantly increased HIF‐1α expression in nuclear extracts of the three lung cancer cells. Bcl‐2/E1B‐nineteen kilodalton interacting protein (BNIP3) plays an important role in the hypoxia‐induced autophagic death of cancer cells.^27^, ^28^ The mechanism by which hypoxia stimulates the expression of BNIP3 most likely involves the direct activation of HIF‐1α.^29^ MAC upregulated BNIP3 expression in a time‐dependent manner (Figure 5B). These results suggest that MAC‐stimulated HIF‐1α and BNIP3 expression is associated with the autophagic activity of MAC.

To determine the relationship between autophagy and HIF‐1α expression, autophagy in the presence of MAC was compared with that in the presence of CoCl_2_ (mimic hypoxia) and EBSS media (amino acid deprivation). Microscopic analysis of cell morphology showed that MAC, CoCl_2_, and EBSS increased vesicle formation. Acridine orange staining showed that MAC, CoCl_2_, and EBSS induced autophagosome vesicle formation. In transfected A549 cells with GFP‐LC3, moreover, MAC, CoCl_2_, and EBSS increased the formation of GFP‐LC3 puncta, a marker that allows for the assessment of autophagic flux (Figure 5C). Autophagosome formation was increased by hypoxia mimic, suggesting that HIF‐1α expression induces autophagy.

Next, the protein expression of autophagy marker genes was analysed in cells cultured in under MAC, CoCl_2_, and EBSS‐containing media. MAC and CoCl_2_ upregulated LC3‐II, Beclin1, ATG7, and BNIP3 in the different lung cancer cells (Figure 5D). However, EBSS‐containing media weakly induced LC3‐II expression and had no effect on the other factors. MAC and CoCl_2_ upregulated HIF‐1α expression, whereas EBSS treatment had no effect on HIF‐1α expression (Figure 5E). These results indicated that MAC induces HIF‐1α expression and autophagy similar to the hypoxia environment.

### 3.6 MAC promotes autophagy by regulating AMPK/HIF‐1α expression in lung cancer cells

**Figure 6:**
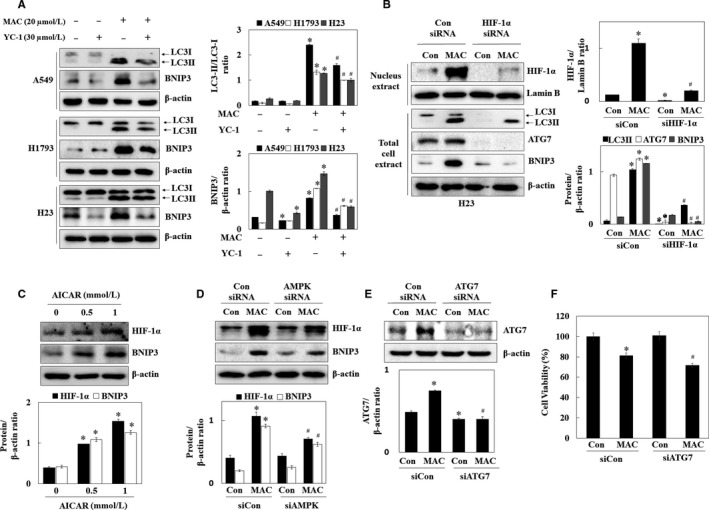
HIF‐1α‐induced autophagy was mediated by the modulation of BNIP3 expression. (A) Cells were treated with MAC (20 μmol/L) and YC‐1 (30 μmol/L) for 12 h and analysed by western blotting with antibodies against LC3 and BNIP3. (B) H23 cells were transfected with control siRNA or HIF‐1α siRNA, and then treated with MAC (20 μmol/L) for 6 or 12 h. Cells were analysed by western blotting with antibodies against HIF‐1α, LC3, ATG7, and BNIP3. Lamin B and β‐actin were used as loading controls. (C) A549 cells were treated with 0.5 and 1 mmol/L AICAR and analysed by western blotting with antibodies against HIF‐1α and BNIP3. β‐actin was used as a loading control. (D) A549 cells were transfected with control siRNA and HIF‐1α siRNA, treated with MAC (20 μmol/L for 6 and 12 h). Cells were analysed by western blotting with antibodies against HIF‐1α and BNIP3. β‐actin was used as a loading control. Densitometric analysis of each protein ratio was performed using ImageJ. (E) A549 cells were transfected with control siRNA or ATG7 siRNA, and then treated with MAC (20 μmol/L) for 12 h. Cells were analysed by western blotting for the expression of ATG7. β‐actin was used as a loading control. (F) Cells were treated with MAC (20 μmol/L) in ATG7‐transfected A549 cells for 24 h, after which cell viability was measured using the MTT assay. The data represent mean ± SE of three independent experiments (*P < 0.05 vs control and ^#^ *P* < 0.05 vs MAC)

To determine whether MAC‐induced autophagy by affecting HIF‐1α expression in lung cancer, the effect of YC‐1 (HIF‐1α inhibitor) on autophagy‐related gene expression was examined. YC‐1 downregulated BNIP3 in A549 and H23 cells, suggesting that HIF‐1α regulates BNIP3 expression in the normoxia condition. MAC upregulated the expression of both LC3 and BNIP3 in the same manner. YC‐1 inhibited the MAC‐induced LC3‐II/LC3‐I ratio and BNIP3 expression (Figure 6A). To determine whether MAC‐induced autophagy was mediated by the regulation of HIF‐1α expression, cells were transfected with siRNA, targeting HIF‐1α. As shown in Figure 6B, MAC‐induced HIF‐1α expression in the control siRNA transfected cells, whereas this effect was suppressed by HIF‐1α siRNA. Transfection with HIF‐1α siRNA substantially abolished the effect of MAC on upregulating autophagy‐associated proteins including LC3, ATG7, and BNIP3. These results suggest that MAC induces autophagy by upregulating HIF‐1α expression.

Previously, we showed that MAC stabilizes HIF‐1α by activating AMPK. To determine whether MAC‐induced autophagy mediated by the regulation of HIF‐1α expression was associated with AMPK activation, the regulatory effect of AMPK on HIF‐1α, and BNIP3 expression was examined using AICAR and AMPK siRNA. As shown in Figure 6C and D, AICAR upregulated HIF‐1α and BNIP3 in a dose‐dependent manner. In AMPK siRNA transfected cells, MAC weakly upregulated HIF‐1α and BNIP3. These results indicated that MAC induces autophagy through AMPK‐dependent and AMPK‐independent mechanisms of MAC‐induced HIF‐1α signalling pathway.

To elucidate the role of selective MAC‐induced autophagy, autophagy was inhibited using the ATG7 siRNA. After the exposure of cells to MAC (20 μmol/L) in the control siRNA‐ or ATG7 siRNA‐transfected cells, the ATG7 protein expression and cytotoxic effects were assessed. Transfection of cells with ATG7 siRNA significantly suppressed the MAC‐induced ATG7 expression in A549 cells (Figure 6E). Inhibition of basal autophagy by ATG7 siRNA was not change cellular viability. MAC at 20 μmol/L in the control siRNA‐transfected cells decreased cell viability by approximately 19%, whereas treatment of cells with MAC in the ATG7 siRNA‐transfected cells led to approximately 28% decrease in cell viability (Figure 6F). These results indicate that inhibition of autophagy sensitizes lung cancer cells to MAC‐induced cytotoxicity, and the combination treatments with autophagy inhibitors might improve the cytotoxic effects of MAC alone.

## 4 DISCUSSION

Autophagy is a lysosome‐mediated degradation mechanism under various stress conditions, such as the high temperatures, starvation conditions, and hypoxic condition.^30^, ^31^ The autophagy process starts with the formation of autophagosomes, which fuse with lysosomes to become autolysosomes and then remove damaged protein and organelles through material recycling, to promote long‐life.^32^, ^33^, ^34^ It was recently reported that autophagy plays an important role in the processes of immunity, infection, inflammation, and tumour formation.^35^, ^36^, ^37^, ^38^, ^39^ In the present study, the autophagic effects of ASC, AS‐6, and MAC in lung cancer cells were investigated. First, the three chemicals were assessed for their induction of autophagy with LC3 expression. ASC did not affect LC3‐II expression, but AS‐6 and MAC significantly increased LC3‐II expression in A549 cells (Figure 1). In the previous study, AS‐6 induced autophagy through ER stress by increasing the essential autophagic proteins Beclin1, ATG5, and LC3‐II in liver carcinoma cells.^40^ However, MAC increased the LC3‐II expression more than the AS‐6. As MAC has a 4‐O‐methyl group to be substituted for the 4‐O‐hydroxyl group of ASC, the methyl group of MAC is important for the activation of autophagy.^41^

Autophagy was related to many members of the ATG family in various stages of autophagosome formation. We investigated the expression of LC3 and Beclin1, which used as autophagy markers, for assessing the autophagy induction by MAC in lung cancer cells. MAC downregulated LC3‐II and Beclin1 (Figure 3). ATG gene family is involved in vesicle formation, processing, and maturation. The E1 ligase ATG7 plays a central role in the elongation of the autophagosomal vesicles, an essential process for damaged‐protein loading, but it also functions in ubiquitin‐like reactions involving the lipidation of LC3 and the conjugation of the ATG5 band ATG12, which is essential for lysosomal fusion.^42^ We found that MAC increased ATG7 expression in a time‐dependent manner. These results suggest that MAC induces autophagy through the ATG family, including LC3, Beclin1, and ATG7.

According to the previous study, AMPK activation is a cellular sensor for the energy balance status, and suppresses cell growth and proliferation in the tumour survival pathway. Considering that AMPK is inactive in the high‐glucose condition, mTOR may be one of the major targets of AMPK signalling. The mTOR signalling plays an essential role in carcinogenesis, which is attributable to its functions in the regulation of growth and metabolism.^43^ In this study, the phosphorylation of AMPK and TSC2, AMPK‐dependent downstream signals, was increased by MAC in lung cancer cells. MAC decreased mTOR phosphorylation only in A549 cell, but mTOR phosphorylation was not changed in H1793 and H23 cells. Although the mTOR phosphorylation, a key factor in protein synthesis, was not decreased by MAC, activation of Raptor, which consists of mTORC1 with mTOR, was decreased by MAC‐induction Raptor phosphorylation in lung cancer cells. In addition, MAC decreased the phosphorylation of P70S6K and 4EBP1. These results suggest that the MAC upregulated AMPK and downregulated mTORC1 stimulates the autophagy process. In addition, we found that AICAR increased AMPK signalling and LC3‐II protein expression. However, the MAC‐induced LC3‐II expression was not completely suppressed in AMPK siRNA transfected cells. Because the process of autophagy was stimulated by many stimulation, such as starvation, ROS, and hypoxia stress,^31^ we focused on hypoxia stress by MAC in lung cancer cells. HIF‐1 has been shown to be overexpressed in hypoxia and to be an important transcription factor for adapting the hypoxia response, and it is composed of a labile HIF‐1α subunit and a stable HIF‐1β subunit.^44^ Under hypoxia conditions, because it is left out by hydroxylation and ubiquitination, HIF‐1α is stabilized and is translocated to the nucleus and forms a heterodimer with HIF‐1β.^45^ It was speculated that the hypoxia‐specific proteins BNIP3 and HIF‐1α were related to the increase of the autophagy‐associated proteins. BNIP3 is stimulated by hypoxia and induces autophagy by weakening the binding of the Bcl‐2/Beclin1 complex.^10^ Hypoxia also induced autophagy by the upregulation of ATG5 and ATG7.^46^ MAC increased BNIP3, Beclin1 and ATG7 expression, and HIF‐1α nuclear translocation (Figure 5). The low levels of oxygen and nutrients enhance autophagy signalling in the tumour microenvironment.^47^ Therefore, the autophagic activities under the treatment of MAC, CoCl_2_ (mimic hypoxia), or EBSS media (amino acid deprivation) were compared. In Figure 5, all the conditions induced autophagosome formation, but EBSS selectively increased LC3‐II. According to a previous study, EBSS increased the phosphorylation of Beclin1 without the regulation of Beclin1 expression.^48^ Our results also showed that the EBSS medium did not increase the Beclin1 expression. As CoCl_2_ can mimic the hypoxia condition by replacing the prolyl hydroxylase, CoCl_2_ increased the HIF‐1α expression similar to the MAC treatment. As expected, the expressions of LC3‐II, Beclin1, ATG7, and BNIP3 were increased by CoCl_2_, which suggest that HIF‐1α expression plays an important role in autophagy. In addition, YC‐1 significantly inhibited MAC‐induced LC3‐II and BNIP3 expression in H1793 and A549 cells. HIF‐1α siRNA induced the downregulation of the MAC‐induced expression of autophagy marker proteins, which suggests MAC‐induced autophagy though HIF‐1α expression. Although the mTOR signalling pathway, a key factor in protein synthesis, was decreased by MAC, MAC increased the synthesis of the HIF‐1α protein. These results suggest that the downregulation of mTORC1 by MAC stimulates the autophagy process without any relation with HIF‐1α expression. Furthermore, we found that MAC‐stimulated HIF‐1α and BNIP3 expression was regulated by AMPK activation.

**Figure 7:**
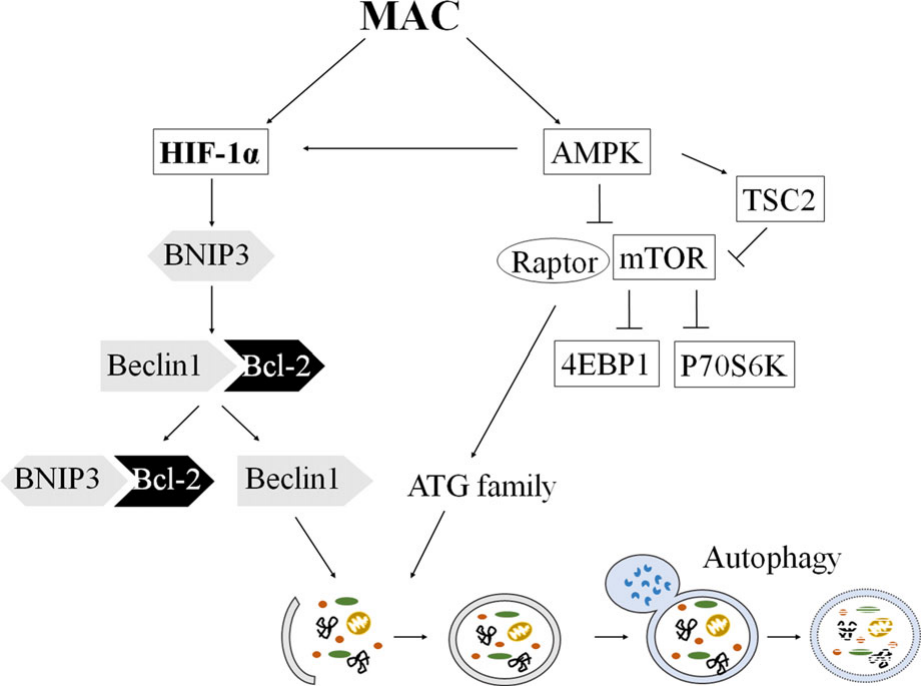
Schematic model of the stimulation of autophagy by MAC in lung cancer cells. MAC induced autophagosome formation by upregulating LC3‐II and ATG7 via the AMPK/mTORC1 signalling pathway. MAC‐stimulated HIF‐1α enhanced autophagy thought BNIP3 and Beclin1. In addition, MAC‐activated AMPK was related to the upregulation of HIF‐1α expression

In conclusion, the mechanism of MAC‐induced autophagy in various lung cancer cells was found for the first time in this study. The autophagic effects of MAC were mediated by the AMPK/mTORC1 signalling pathway and the upregulation of HIF‐1α protein expression (Figure 7). The findings of this study imply the role of MAC in determining the response of lung cancer cells. Further studies are expected to confirm the relationship between the autophagic activity of MAC and the death of lung cancer cells.

## CONFLICT OF INTEREST

The authors confirm that there are no conflicts of interest.
